# Antitumor Evaluation of Rhenium-188 and Paclitaxel Co-Delivered via Thermosensitive Hydrogel in a Hepatocellular Carcinoma Animal Model

**DOI:** 10.3390/ijms27020775

**Published:** 2026-01-13

**Authors:** Ying-Hsia Shih, Cheng-Liang Peng, Ping-Fang Chiang, Chun-Tang Chen

**Affiliations:** Department of Isotope Application Research, National Atomic Research Institute, P.O. Box 3-27, Longtan, Taoyuan 32546, Taiwan; shihys@nari.org.tw (Y.-H.S.);

**Keywords:** unresectable HCC, rhenium-188, paclitaxel, hydrogel

## Abstract

Hepatocellular carcinoma (HCC) remains one of the most common malignancies worldwide and a leading cause of cancer-related mortality. Current treatment options for advanced or unresectable HCC have limited efficacy and are often associated with systemic toxicity. In this study, a multifunctional, thermosensitive hydrogel-based delivery system was developed to enhance localized treatment of HCC. This system incorporates rhenium-188 sulfur colloid (^188^Re-colloid), a β-emitting radiotherapeutic agent, and paclitaxel (PTX)-loaded micelles within a biodegradable PCL-PEG-PCL hydrogel matrix. The formulation enables in situ gelation at physiological temperatures, providing sustained release and prolonged retention of therapeutic agents at the tumor site. Physicochemical characterization confirmed the structural integrity and injectability of the formulations, while in vivo biodistribution studies in a murine hepatic tumor model demonstrated enhanced intratumoral accumulation and reduced systemic dispersion. The combined chemo-radiotherapeutic platform showed potential for improved therapeutic efficacy through synergistic action, offering a promising minimally invasive strategy for treating unresectable hepatocellular carcinoma.

## 1. Introduction

Hepatocellular carcinoma (HCC) remains one of the most prevalent malignancies worldwide and represents a leading cause of cancer-related mortality, particularly in regions with a high incidence of chronic liver disease [1,2,3]. Despite advances in surgical techniques, locoregional therapies, and systemic treatments, the prognosis for patients with advanced or unresectable HCC remains poor. Limitations such as insufficient drug accumulation within tumors and dose-limiting systemic toxicity continue to compromise therapeutic outcomes, highlighting the need for more effective localized treatment strategies.

The current therapeutic landscape for HCC comprises multiple locoregional and systemic treatment options, including surgical resection, transarterial chemoembolization (TACE), systemic chemotherapeutic regimens, microwave ablation, radiofrequency ablation (RFA), and internal radionuclide-based radiotherapeutic approaches [4,5,6,7,8]. Among these modalities, surgical resection is generally associated with the most favorable long-term survival outcomes; however, its clinical applicability is limited to a relatively small proportion of patients, as many present with advanced disease, unfavorable tumor location, or compromised hepatic function at diagnosis [9,10]. For patients with unresectable or metastatic HCC, systemic chemotherapy and targeted agents such as tyrosine kinase inhibitors or immune checkpoint inhibitors are often employed as frontline treatments. However, despite improvements in overall survival achieved by these regimens, the therapeutic benefit remains modest and is frequently accompanied by substantial treatment-related adverse effects, including high rates of severe toxicity [11]. These limitations highlight an unmet clinical need for alternative treatment approaches capable of achieving effective local tumor control while minimizing systemic adverse effects [12]. Consequently, localized and targeted therapeutic strategies, including intratumoral drug delivery and radionuclide-based interventions, have attracted increasing attention as promising modalities for improving treatment outcomes in HCC. Consequently, localized and targeted therapeutic approaches such as intratumoral drug delivery and radionuclide-based interventions have attracted increasing attention as promising modalities for enhancing treatment efficacy and minimizing systemic toxicity in unresectable HCC [7,8,13].

Radionuclide-based therapy, particularly approaches employing β-emitting isotopes such as rhenium-188 (^188^Re), has emerged as a promising treatment option for hepatocellular carcinoma owing to its favorable physical characteristics [14,15]. The isotope possesses a relatively short physical half-life of approximately 16.9 h and emits high-energy β-particles, allowing efficient tumor cell irradiation while limiting radiation exposure to surrounding normal tissues. To further enhance therapeutic efficacy and localize radiation delivery, ^188^Re has been integrated into a variety of carrier systems designed to prolong intratumoral retention, including colloidal formulations [14], thermosensitive hydrogels [16], particulate delivery systems [17,18,19], and micelle-based composites [16]. In our previous studies, thermosensitive hydrogel-based delivery of ^188^Re formulations demonstrated improved intratumoral retention and favorable biodistribution profiles. However, whether such enhanced local retention could be translated into a meaningful survival benefit remains unclear. Therefore, the present study was designed to systematically evaluate the therapeutic efficacy of a multifunctional ^188^Re-colloid/PTX-micelle hydrogel, with particular emphasis on long-term tumor control and survival outcomes.

The present study sought to develop and systematically assess a series of ^188^Re-based therapeutic platforms for the treatment of unresectable HCC. The investigated formulations included ^188^Re sulfur colloid [16], a thermosensitive hydrogel encapsulating ^188^Re-colloid, and a multifunctional composite hydrogel co-loaded with ^188^Re-colloid and paclitaxel (PTX)-encapsulated micelles. The ^188^Re sulfur colloid was prepared by reduction of rhenium-188 using stannous chloride, resulting in the formation of suspended sulfur colloid particles capable of serving as radionuclide carriers with both therapeutic and diagnostic potential. To improve intratumoral retention and reduce systemic dissemination of radioactivity, the ^188^Re-colloid was incorporated into a thermosensitive hydrogel matrix. Furthermore, to enable combined chemo-radiotherapeutic intervention, PTX-loaded micelles—an established chemotherapeutic strategy for hepatocellular carcinoma—were co-encapsulated within the same hydrogel system, generating an integrated ^188^Re-colloid/PTX-micelle hydrogel platform. Comprehensive physicochemical characterization was performed for each formulation, followed by in vivo evaluation in a murine subcutaneous hepatic tumor model to investigate tumor retention, biodistribution profiles, and overall therapeutic potential.

Thermosensitive hydrogels have attracted considerable attention as injectable drug delivery systems due to their ability to undergo temperature-triggered phase transitions [20,21]. In this study, a biodegradable triblock copolymer, poly(ε-caprolactone)-poly(ethylene glycol)-poly(ε-caprolactone) (PCL–PEG–PCL), was employed as the hydrogel matrix [16]. This polymer remains in a liquid state at low temperatures and rapidly transforms into a semi-solid gel at physiological temperatures [22]. Following intratumoral or subcutaneous administration, the hydrogel forms an in situ depot that persists at the injection site for approximately 1–2 weeks, enabling sustained local release of therapeutic agents. Owing to its injectability and localized gel formation, this hydrogel-based platform offers a minimally invasive approach that may reduce systemic drug exposure and improve treatment convenience [23,24,25].

**Figure 1:**
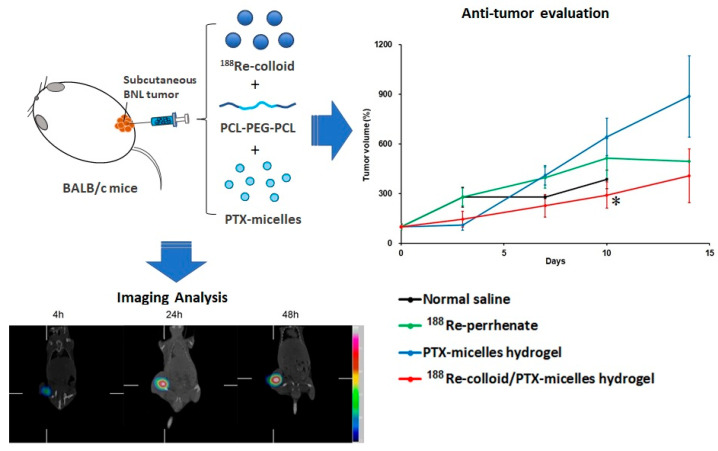
Schematic illustration of the experimental design for intratumoral administration of ^188^Re-colloid/PTX-micelle hydrogel in BNL-bearing BALB/c mice. * *p* < 0.05.

In this study, a thermosensitive hydrogel was developed as a dual-delivery platform for both ^188^Re-colloid and paclitaxel (PTX)-loaded micelles, with the aim of achieving localized and sustained release of therapeutic agents at the tumor site (Figure 1). BNL-bearing BALB/c mice were randomly assigned to four treatment groups: normal saline (control), ^188^Re-perrhenate (^188^ReO_4_^−^), PTX-micelle hydrogel, and ^188^Re-colloid/PTX-micelle hydrogel, all of which were administered via intratumoral injection. In vivo evaluations, including Micro-SPECT/CT imaging, biodistribution analysis, tumor volume monitoring, and survival analysis, were performed using a murine hepatic tumor model to assess intratumoral retention, radiotracer distribution, and therapeutic efficacy.

## 2. Results and Discussion

### 2.1. Formation and Characterization of ^188^Re-Colloid/PTX-Micelle Hydrogel

**Figure 2:**
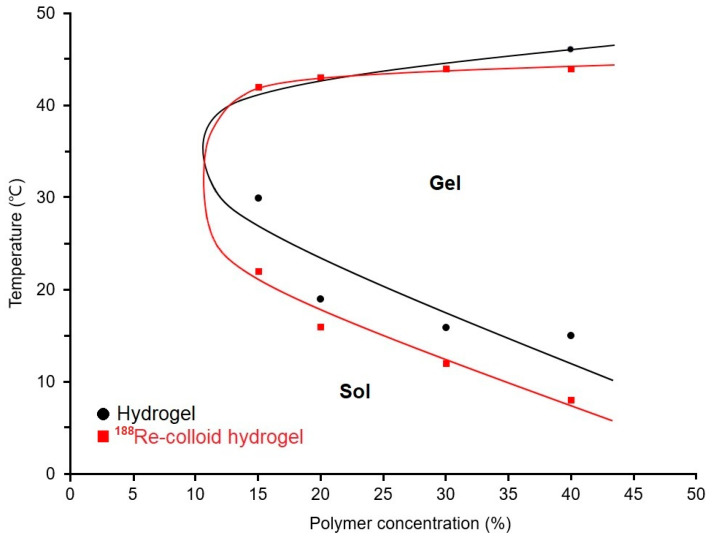
Temperature-dependent sol–gel–sol phase transition behavior of the PCL–PEG–PCL hydrogel system; representative data are shown and statistical analysis was not performed because the figure serves to demonstrate qualitative trends.

The phase behavior of the PCL–PEG–PCL triblock copolymer in aqueous solution was strongly influenced by both polymer concentration and temperature. At concentrations below 15% (*w*/*v*), the formulation remained in a fluid state across the entire temperature range investigated, suggesting inadequate micellar assembly to form a three-dimensional network. In contrast, formulations with polymer concentrations of 20% (*w*/*v*) or higher displayed a distinct and reversible sol–gel–sol transition. Gelation occurred at intermediate temperatures close to physiological conditions (approximately 30–40 °C), while a transition back to the sol state was observed at elevated temperatures exceeding 42 °C, consistent with the thermo-responsive nature of amphiphilic block copolymers [26,27]. Incorporation of ^188^Re-colloid into the hydrogel matrix resulted in a noticeable reduction in the sol–gel transition temperature across all evaluated polymer concentrations (15–40% *w*/*v*), as illustrated in Figure 2. This shift suggests that the presence of the radionuclide colloid may enhance micellar interactions or promote hydrophobic aggregation, thereby facilitating gel formation at lower temperatures. Notably, the gel–sol transition at higher temperatures was minimally affected by the addition of ^188^Re-colloid. Based on the favorable gelation behavior and thermal responsiveness, a polymer concentration of 25% (*w*/*v*) was selected for subsequent in vivo experiments. The resulting thermosensitive hydrogel enabled injection in the sol state followed by rapid gelation under physiological conditions, while maintaining uniform distribution of both ^188^Re-colloid and PTX-loaded micelles within the polymer network [28]. This structural homogeneity indicates good compatibility between the hydrogel matrix and the incorporated therapeutic components, supporting its suitability as a localized delivery platform for intratumoral administration. The radiolabeling efficiency of the ^188^Re-colloid exceeded 95%, in agreement with previously reported preparation methods and comparable PCL–PEG–PCL-based systems [16].

**Figure 3:**
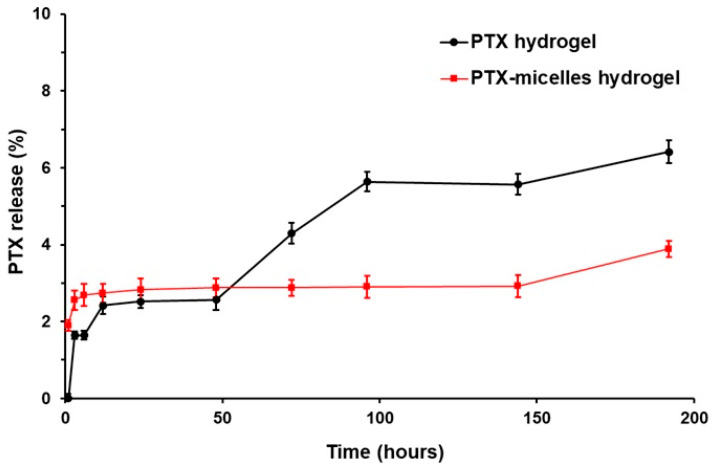
Paclitaxel (PTX) release profiles of the PTX hydrogel and the PTX-micelle–incorporated hydrogel formulations, with data shown as mean ± SD from three independent experiments; statistical analysis was not performed as the purpose was to demonstrate release trends rather than compare groups quantitatively.

The release behavior of paclitaxel from the hydrogel-based formulations was examined to assess drug retention characteristics. As shown in Figure 3, the PTX-micelle-incorporated hydrogel demonstrated minimal drug diffusion, with cumulative paclitaxel release remaining below 10% throughout the entire observation period. This limited release indicates that most of the encapsulated PTX was effectively retained within the hydrogel network, reflecting a sustained-release profile. A comparative analysis between the PTX-loaded hydrogel and the PTX-micelle-loaded hydrogel further highlighted the impact of micellar incorporation on release kinetics. The micelle-containing formulation exhibited a markedly reduced release rate relative to the non-micellar PTX hydrogel, underscoring the enhanced drug confinement achieved through micelle–hydrogel integration, in agreement with previously reported observations [29,30].

### 2.2. Micro-SPECT/CT Imaging of ^188^Re-Colloid/PTX-Micelle Hydrogel

**Figure 4:**
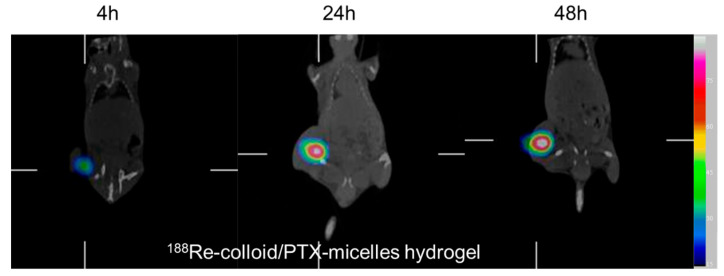
Micro-SPECT/CT images showing intratumoral distribution of ^188^Re-colloid/PTX-micelle hydrogel in BNL tumor-bearing mice at 4, 24, and 48 h following intratumoral injection; statistical analysis was not applied because the images are intended for qualitative visualization.

Micro-SPECT/CT imaging was performed in the present study to investigate the intratumoral localization and temporal distribution of radioactivity following intratumoral administration of the ^188^Re-colloid/PTX-micelle hydrogel. Serial imaging demonstrated pronounced and persistent radioactivity confined within the tumor region at 4, 24, and 48 h post-injection (Figure 4), with the spatial distribution remaining largely restricted to the injection site, indicating effective intratumoral retention over an extended period. For comparison, the in vivo behavior of freely diffusible ^188^Re-perrhenate following intratumoral administration has been previously reported. In those studies, ^188^Re-perrhenate exhibited rapid loss of localized signal, with widespread systemic distribution observed as early as 1 h post-injection [15,16]. This distribution pattern is attributed to the low molecular weight and high aqueous solubility of ^188^Re-perrhenate, which facilitates rapid diffusion from the tumor microenvironment and poor site-specific retention. Collectively, these observations highlight a marked difference in intratumoral retention between the two formulations. Encapsulation of ^188^Re-colloid within the thermosensitive hydrogel substantially prolonged radionuclide residence at the tumor site compared with freely diffusible ^188^Re-perrhenate, underscoring the importance of hydrogel-based confinement for improving the therapeutic index of localized radionuclide therapy by maximizing tumor exposure while minimizing radiation delivery to surrounding normal tissues.

### 2.3. Quantitative Biodistribution of ^188^Re-Colloid/PTX-Micelle Hydrogel

**Figure 5:**
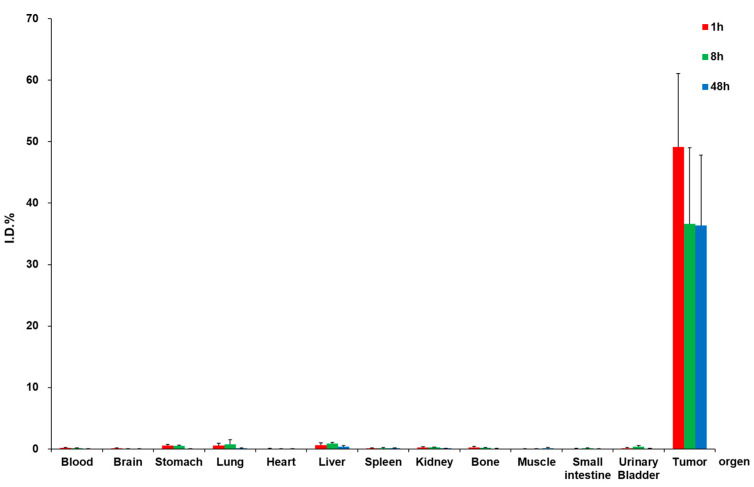
Quantitative biodistribution of ^188^Re-colloid/PTX-micelle hydrogel in BNL tumor-bearing mice at 1, 8, and 48 h post-intratumoral injection (*n* = 3 per time point) with data shown as mean ± SD; statistical analysis was not performed since the aim was to illustrate temporal distribution trends.

Quantitative biodistribution and Micro-SPECT/CT imaging were performed using separate cohorts of mice to evaluate the intratumoral retention of ^188^Re-colloid/PTX-micelle hydrogel in BNL tumor-bearing mice. Biodistribution analysis at 1, 8, and 48 h and imaging at 4, 24, and 48 h showed that the majority of the injected radioactivity remained localized within the tumor at all time points. The selected intervals were guided by the physical half-life of ^188^Re (~17 h) and previous preclinical and clinical SPECT studies, capturing both early distribution and prolonged retention while avoiding repeated anesthesia or radiation exposure [18,19,31]. As summarized in Table 1 and illustrated in Figure 5, the majority of the injected radioactivity remained localized within the tumor at all examined time points.

Tumor-associated uptake accounted for approximately 49.2 ± 11.9% of the injected dose at 1 h and remained at 36.6 ± 12.4% and 36.3 ± 11.5% at 8 and 48 h post-injection, respectively, demonstrating sustained intratumoral retention over time. In contrast, radioactivity levels in non-target organs were comparatively low throughout the study period. Moderate uptake was observed in the liver and lungs, while radioactivity detected in the kidneys, urinary bladder, and blood remained minimal, indicating limited systemic leakage and renal clearance following intratumoral administration. Other organs, including the spleen, heart, brain, bone, and gastrointestinal tract, exhibited only low levels of radioactivity. Consistent with the Micro-SPECT/CT imaging findings, the biodistribution results further confirm prolonged confinement of the radionuclide at the tumor site for up to 48 h post-injection. This favorable distribution profile is likely attributable to the thermosensitive gelation of the PCL–PEG–PCL copolymer, which forms a semi-solid depot at physiological temperature, thereby immobilizing the ^188^Re-colloid within the tumor mass. Collectively, these results demonstrate that the hydrogel formulation effectively limits off-target exposure while maintaining high intratumoral radioactivity, supporting its suitability for localized radionuclide therapy [25].

### 2.4. Antitumor Efficacy and Tumor Growth Inhibition

**Figure 6:**
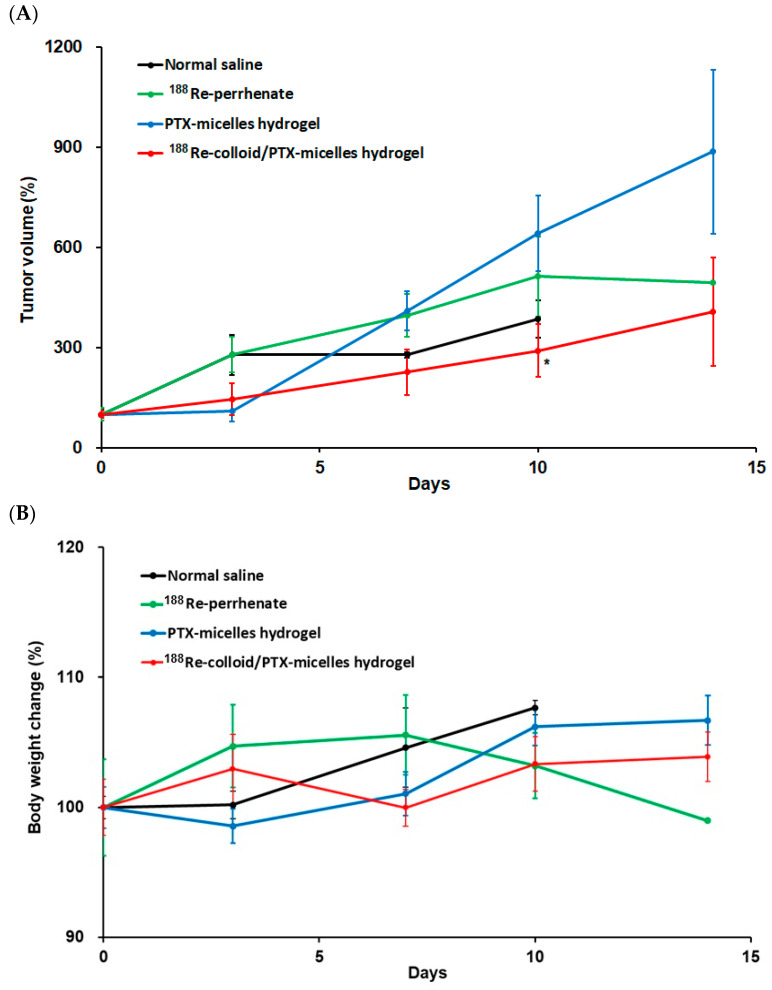
Anti-tumor effect of ^188^Re-colloid/PTX-micelle hydrogel. Tumor volume (**A**) and body weight changes (**B**) in BNL Tumor-Bearing Mice post intratumoral injection following normal saline, ^188^Re-perrhenate, PTX-micelles hydrogel and ^188^Re-colloid/PTX-micelle hydrogel. (*n* = 3 each group); data are presented as mean ± SD, and statistical significance was evaluated using Student’s *t*-test. Statistical significance is indicated as follows: * *p* < 0.05 vs. control and PTX-micelle hydrogel.

Antitumor efficacy and survival-related outcomes were evaluated in BNL tumor-bearing mice following intratumoral administration of different treatment formulations. Mice were treated with normal saline (control), ^188^Re-perrhenate, PTX-micelle hydrogel, or the combined ^188^Re-colloid/PTX-micelle hydrogel. Tumor growth was monitored longitudinally to assess therapeutic response (Figure 6A), and changes in body weight were recorded throughout the study as an indicator of systemic tolerance (Figure 6B). At day 10 following treatment, tumor volume analysis revealed divergent growth behaviors among the experimental groups. The normal saline group reached a tumor volume of 278.77 ± 55.91%; however, only two animals remained at this time point, limiting the reliability of direct quantitative comparison with other groups. In contrast, mice receiving monotherapy exhibited pronounced tumor progression, with tumor volumes increasing to 513.73 ± 120.20% in the ^188^Re-perrhenate group and 642.81 ± 113.99% in the PTX-micelle hydrogel group, indicating limited antitumor efficacy when either modality was administered alone. Notably, treatment with the combined ^188^Re-colloid/PTX-micelle hydrogel resulted in a substantially attenuated increase in tumor volume (291.54 ± 79.09%), demonstrating improved tumor growth control relative to monotherapy groups. Although reduced animal numbers in the control group constrained robust statistical comparison at later time points, tumor growth in the combination group was significantly lower than that observed in the PTX-micelle hydrogel group at day 10 (*p* < 0.05), supporting the enhanced therapeutic benefit of localized chemo-radiotherapy. Furthermore, mice treated with ^188^Re-perrhenate alone exhibited a gradual decline in body weight over the study period, suggesting a greater systemic burden compared with hydrogel-based formulations. In contrast, animals receiving the ^188^Re-colloid/PTX-micelle hydrogel maintained relatively stable body weights throughout the study, indicating minimal adverse effects. These findings suggest that the combined hydrogel formulation not only enhances tumor growth inhibition but also provides improved systemic tolerability, supporting a potential synergistic therapeutic benefit.

### 2.5. Survival Analysis

**Figure 7:**
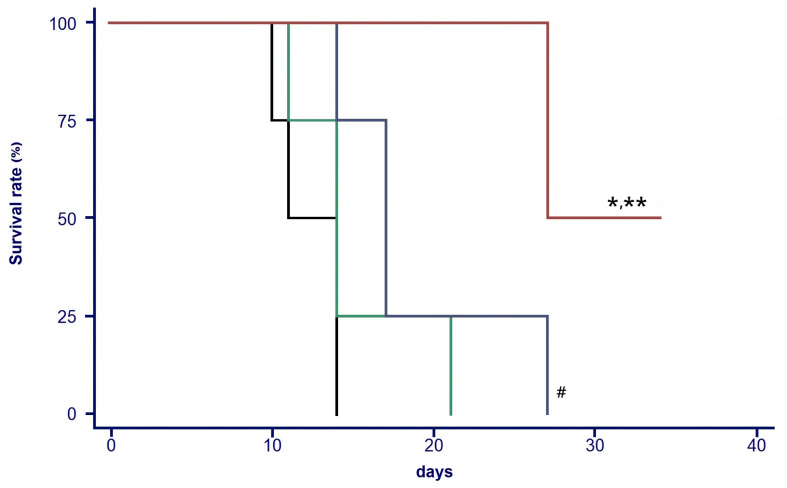
Survival of BNL tumor-bearing mice following intratumoral administration of different treatments. Groups included normal saline (━), ^188^Re-perrhenate (━), PTX-micelles hydrogel (━) and ^188^Re-colloid/PTX-micelle hydrogel (━). Data are shown for *n* = 4 mice per group. Survival curves were analyzed using the Kaplan–Meier method, and differences between groups were evaluated by the log-rank test. Statistical significance is indicated as follows: # *p* < 0.05 vs. control; * *p* < 0.05 vs. PTX-micelle hydrogel; ** *p* < 0.01 vs. control and ^188^Re-perrhenate.

In the survival analysis, mice receiving normal saline exhibited the poorest outcomes, with individual survival times of 10, 11, 14, and 14 days, reflecting rapid tumor progression in the absence of therapeutic intervention. The group treated with ^188^Re-perrhenate showed modest improvement, with survival durations of 11, 14, 14, and 21 days, indicating limited benefit from radionuclide therapy alone. Similarly, mice administered the PTX-micelle hydrogel demonstrated slightly prolonged survival, with individual survival times of 14, 17, 17, and 27 days, suggesting partial therapeutic efficacy of chemotherapy delivered via the hydrogel matrix (Figure 7).

In contrast, the ^188^Re-colloid/PTX-micelle hydrogel group achieved the most favorable survival outcomes among all treatment groups. Individual survival times in this cohort were 27, 27, 34, and 34 days, with two mice surviving until the experimental endpoint and one mouse exhibiting complete tumor regression. These findings highlight the enhanced therapeutic benefit of the combined chemo-radiotherapeutic strategy, likely attributable to sustained intratumoral co-delivery of both agents.

Survival analysis revealed statistically significant differences among the treatment groups. Mice treated with the PTX-micelle hydrogel exhibited significantly prolonged survival compared to the control group (*p* < 0.05). Moreover, the combined ^188^Re-colloid/PTX-micelle hydrogel group demonstrated significantly improved survival outcomes compared to the control group and the ^188^Re-perrhenate group (*p* < 0.01). Importantly, the combined ^188^Re-colloid/PTX-micelle hydrogel group showed a further significant survival benefit over the PTX-micelle hydrogel group alone (*p* < 0.05), suggesting a synergistic therapeutic effect of radiotherapy and chemotherapy when co-delivered via the hydrogel platform.

Although the present study demonstrates enhanced antitumor efficacy of the combined ^188^Re-colloid/PTX-micelle hydrogel compared with monotherapy controls, the absence of a hydrogel-embedded ^188^Re-colloid–only group represents a limitation in fully delineating the individual contributions of radionuclide immobilization and chemotherapeutic synergy. This design choice reflects the primary aim of the current work to evaluate therapeutic outcomes using clinically relevant comparator groups while minimizing additional animal usage. Future studies incorporating expanded control formulations will be valuable for further elucidating the mechanistic basis of the observed chemo-radiotherapeutic effects.

In comparison with our previous studies, several important distinctions should be emphasized. While earlier work has already demonstrated intratumoral retention and quantitative biodistribution characteristics of ^188^Re-based hydrogel or particulate systems, those studies primarily focused on distribution behavior and short-term therapeutic response. In contrast, the present study extends this framework by integrating PTX-loaded micelles as the chemotherapeutic component and systematically evaluating long-term therapeutic benefit through survival analysis in addition to tumor growth inhibition. This expanded assessment provides a more comprehensive evaluation of treatment efficacy and tolerability within a multifunctional hydrogel platform [16,19].

Collectively, these findings underscore the therapeutic advantage of combining radionuclide therapy with controlled chemotherapeutic delivery in the BNL hepatoma model. Whereas monotherapies, including PTX-micelle hydrogel or ^188^Re-perrhenate alone, produced only limited survival benefits, co-encapsulation of both modalities within a thermosensitive hydrogel resulted in a marked prolongation of survival. This improvement is likely attributable to sustained and localized co-delivery of radiotherapeutic and chemotherapeutic agents at the tumor site, thereby maximizing antitumor efficacy while minimizing systemic exposure [32,33]. The statistically significant differences observed among treatment groups further support the concept that integration of radionuclide therapy with advanced drug delivery systems can enhance in vivo therapeutic outcomes beyond those achievable with single-modality approaches.

Importantly, mice receiving the combined ^188^Re-colloid/PTX-micelle hydrogel demonstrated superior tumor control and prolonged survival, suggesting a synergistic effect between radionuclide therapy and chemotherapy when delivered via a localized, sustained-release hydrogel system. Throughout the study, body weight remained relatively stable across all treated groups, indicating minimal systemic toxicity. These results collectively support the enhanced therapeutic efficacy of the ^188^Re-colloid/PTX-micelle hydrogel formulation, both in terms of tumor growth inhibition and improved survival benefit.

While the present study demonstrates the promising therapeutic potential of the ^188^Re-colloid/PTX-micelle hydrogel, several considerations should be noted when interpreting the results. First, all in vivo experiments were performed using female BALB/c mice. This experimental design was selected to ensure stable tumor establishment and to minimize biological variability; however, potential sex-related differences in tumor behavior and treatment response were not specifically addressed in the current study.

In addition, the number of animals included in each experimental group was limited, as is typical for exploratory preclinical investigations. Although this sample size was sufficient to reveal clear therapeutic trends, larger cohorts may further strengthen statistical robustness. Moreover, while the combined hydrogel formulation exhibited superior efficacy compared with monotherapy controls, the inclusion of a hydrogel-based ^188^Re-colloid–only group could provide additional insight into the individual contributions of radionuclide retention and chemotherapeutic effects.

Finally, a subcutaneous tumor model was employed to facilitate longitudinal monitoring of tumor growth and treatment response. Although this model is widely used and reproducible, it does not fully recapitulate the hepatic microenvironment of orthotopic hepatocellular carcinoma. Furthermore, the observation period was limited to 34 days, and longer-term outcomes were not evaluated. Future studies incorporating orthotopic models, extended follow-up, and expanded experimental groups may help further elucidate the translational potential of this therapeutic strategy.

## 3. Materials and Methods

### 3.1. Preparation of the PCL–PEG–PCL Triblock Copolymer

The poly(ε-caprolactone)–poly(ethylene glycol)–poly(ε-caprolactone) (PCL–PEG–PCL) triblock copolymer, with a molecular weight ratio of 0.75:1:0.75, was synthesized and characterized in accordance with previously reported procedures [16]. For hydrogel preparation, the copolymer was slowly dispersed into deionized water under continuous stirring until a uniform solution was obtained, yielding a final polymer concentration of 25% (*w*/*v*).

The sol–gel transition behavior of the hydrogel was evaluated using a tube inversion method [34]. Copolymer solutions at various concentrations were equilibrated at 4 °C for 10 min, followed by gradual heating. Gelation was defined as the temperature at which samples (15–40% *w*/*v*) ceased to flow upon inversion for at least 1 min. Measurements were performed in triplicate, and mean values were used to generate the phase diagram.

### 3.2. Preparation and Release Evaluation of PTX Hydrogel and PTX-Micelle–Incorporated Hydrogel

Paclitaxel (PTX) release from the PTX-loaded hydrogel and PTX-micelle–incorporated hydrogel was assessed using a dialysis method. Briefly, 1 mL of PTX solution or PTX-micelle suspension (2 mg/mL PTX) was separately incorporated into a 25% (*w*/*v*) hydrogel and sealed in dialysis bags. The preparation of PTX-loaded micelles has been reported previously [35]. At predetermined time points, aliquots of the release medium were collected and first analyzed for radioactivity using a gamma counter (Cobra Series Model 5003, Packard, Prospect, CT, USA). The dialysis bags were maintained in 50 mL of phosphate-buffered saline (PBS; pH ~7.4) preheated to 37 °C under gentle agitation at 300 rpm throughout the experiment. After collection, the samples were stored at −20 °C until analysis. The amount of paclitaxel released was subsequently quantified by high-performance liquid chromatography (HPLC). In addition, the paclitaxel loading in the micelle formulation was verified by HPLC analysis using a validated calibration curve generated from PTX reference standards, confirming a final PTX concentration of 2 mg/mL prior to incorporation into the hydrogel system [36,37]. All experiments were conducted in triplicate, and the results are presented as mean ± standard deviation (SD).

### 3.3. Preparation of ^188^Re-Colloid/PTX-Micelles Hydrogel

A carrier-free ^188^Re-perrhenate (^188^ReO_4_^−^) solution was obtained by elution from a ^188^W/^188^Re generator (National Atomic Research Institute, NARI, Taoyuan, Taiwan). The synthesis of ^188^Re-colloid was carried out using a modified protocol adapted from previously reported methods [16]. In brief, stannous chloride (SnCl_2_, 35 mg) was added to the ^188^Re-perrhenate solution (~370 MBq/mL), and the mixture was incubated at 50 °C for 5–20 min to facilitate reduction and colloid formation. After completion of the reaction, the suspension was allowed to cool to room temperature and subsequently centrifuged at 3000 rpm for 10 min. The supernatant was removed, and the resulting ^188^Re-colloid pellet was resuspended in a PTX-micelle solution prepared according to previously established procedures [19].

The radiolabeling efficiency of the ^188^Re-colloid was determined by instant thin-layer chromatography (ITLC) using silica gel strips as the stationary phase and normal saline as the mobile phase. The distribution of radioactivity along the strips was quantified using a TLC scanner (AR2000, Bioscan, Washington, DC, USA).

For fabrication of the ^188^Re-colloid/PTX-micelle hydrogel, PCL–PEG–PCL triblock copolymer powder was gradually incorporated into the PTX-micelle suspension containing ^188^Re-colloid under controlled temperature conditions. The mixture was cooled to 4 °C to ensure uniform dispersion and subsequently blended with the hydrogel base solution to yield a homogeneous formulation. The final polymer concentration was adjusted to 25% (*w*/*v*), with the radioactivity of ^188^Re set at approximately 370 MBq/mL and the PTX-micelle concentration fixed at 2 mg/mL.

The thermoresponsive sol–gel transition behavior of ^188^Re-colloid hydrogel formulations at concentrations of 15%, 20%, 30%, and 40% (*w*/*v*) was evaluated using a tube inversion method. Gelation was determined by visual inspection of flow behavior upon inversion of the sample tube at increasing temperatures.

### 3.4. Cell Culture

Subcutaneous hepatocellular carcinoma tumors were established using the BNL-Luc cell line, which originates from chemically transformed hepatic epithelial cells of BALB/c mice. Cells were routinely maintained in Dulbecco’s Modified Eagle Medium (DMEM; Gibco BRL, Gaithersburg, MD, USA) supplemented with 10% heat-inactivated fetal bovine serum (FBS; Gibco BRL) and 1% antibiotic–antimycotic solution. Cultures were incubated at 37 °C under humidified conditions with an atmosphere of 5% CO_2_ to support optimal cell growth.

### 3.5. Murine Hepatocellular Carcinoma Xenograft Model

A subcutaneous hepatocellular carcinoma (HCC) model was established using BNL tumor cells. Female BALB/c mice (5–6 weeks old) were obtained from BioLASCO (Ilan, Taiwan) and housed under specific pathogen-free conditions. They were selected for this study because they are immunocompetent and widely used in syngeneic BNL HCC models, which ensures consistent tumor engraftment and minimizes inter-animal variability [38,39]. Mice were subcutaneously inoculated in the right hind limb with 1 × 10^6^ BNL cells suspended in phosphate-buffered saline (PBS). This cell dose was selected based on established protocols, as it reliably produces measurable subcutaneous tumors within 7–10 days, allowing consistent monitoring of tumor growth and reproducible evaluation of antitumor efficacy [19]. Tumor growth was monitored every 2–3 days, and palpable tumors were consistently observed within approximately 7 days following cell inoculation.

All animal experiments were conducted in accordance with the guidelines approved by the Institutional Animal Care and Use Committee (IACUC) of the National Atomic Research Institute (protocol no. 110014). Mice were housed at 21–23 °C with a relative humidity of 50–60% under a 12 h light/12 h dark cycle, with 3–5 animals per cage. Standard laboratory chow and water were provided ad libitum. Euthanasia was performed in accordance with approved IACUC guidelines using gradual carbon dioxide (CO_2_) inhalation until cessation of respiration and cardiac activity was confirmed.

### 3.6. In Vivo Scintigraphy and Ex Vivo Bio-Distribution

Mice bearing tumors of 100–150 mm^3^, a size commonly used in previous preclinical studies where tumors remained measurable and continued to grow over the observation period, enabling reliable detection and meaningful comparison of treatment efficacy [16,19], were intratumorally injected with ^188^Re-colloid/PTX-micelle hydrogel (74 MBq of ^188^Re in 0.1 mL; PTX dose equivalent to 10 mg/kg). For in vivo imaging, a dedicated cohort of mice (n = 3) was used for longitudinal Micro-SPECT/CT scans at 4, 24, and 48 h post-injection. It should be noted that in vivo imaging of freely diffusible ^188^Re-perrhenate was not performed in this study; comparisons to ^188^Re-perrhenate at early time points are based on previously published data [19].

Micro-SPECT/CT imaging was performed using a small-animal SPECT/CT system (XSPECT, Gamma Medica, Northridge, CA, USA) equipped with a low-energy high-resolution collimator. During imaging, mice were anesthetized with isoflurane delivered via inhalation and immobilized to ensure consistent positioning. Following SPECT acquisition, CT scans were immediately performed using an X-ray source operated at 50 kV and 0.4 mA. SPECT images were reconstructed using LumaGEM software (version 5.407), CT images were reconstructed with COBRA Exxim software (version 6.3.39.0), and image fusion was conducted using IDL 6.0 (Research Systems Inc., Boulder, CO, USA).

For biodistribution studies, a separate cohort of mice was used. Animals (n = 3 per time point) were euthanized at 1, 8, and 48 h post-injection. Tumors and major organs were harvested, weighed, and measured for radioactivity using a gamma counter. Biodistribution data were expressed as the percentage of injected dose per organ (%ID).

To evaluate biodistribution and imaging over time, we selected early to late post-injection intervals. The physical half-life of ^188^Re (~17 h) and previous preclinical and clinical SPECT studies guided the choice of 1, 8, and 48 h for biodistribution and 4, 24, and 48 h for Micro-SPECT/CT imaging, capturing both initial distribution and prolonged intratumoral retention [14,18,19,31]. For biodistribution studies, three mice per time point were used, which is consistent with established practices for radiotracer experiments where low inter-animal variability is expected. The primary goal was to characterize temporal distribution and intratumoral retention [40,41].

### 3.7. Antitumor Efficacy and Survival Analysis

Therapeutic studies were initiated when tumor volumes reached 100–150 mm^3^ (designated as day 0). Mice were randomly assigned to four treatment groups (n = 4 per group): (1) normal saline (control), (2) ^188^Re-perrhenate (^188^ReO_4_) solution, (3) PTX-micelle hydrogel, and (4) ^188^Re-colloid/PTX-micelle hydrogel. A total radioactivity dose of 370 MBq was administered for the ^188^Re-containing formulations, and the PTX dose was equivalent to 10 mg/kg. All treatments were delivered via intratumoral injection.

In contrast with in vivo scintigraphy and ex vivo bio-distribution studies, the antitumor efficacy and survival studies involved longitudinal measurements of tumor growth, body weight, and survival, which inherently show greater variability among animals. Therefore, four mice per treatment group were used to provide more reliable assessments of therapeutic effects and survival trends over time, while remaining within ethical and practical constraints of preclinical studies [18,19,40,41].

Tumor size and body weight were measured every 3–4 days. Tumor volume was calculated using the formula: (length × width^2^)/2. Animals were monitored for 34 days, and humane endpoints were defined as tumor length exceeding 20 mm or body weight loss greater than 20% without recovery.

### 3.8. Statistical Analysis

Data are presented as mean ± SD. Comparisons between groups for tumor growth and body weight were performed using Student’s *t*-test, with *p* < 0.05 considered statistically significant. Survival curves were analyzed using the Kaplan–Meier method, and differences between groups were evaluated by the log-rank test, with *p* < 0.05 considered statistically significant. For figures showing representative images or exploratory trends (Figure 1, Figure 2, Figure 3, Figure 4 and Figure 5), statistical analysis was not performed, as these data were intended for qualitative illustration.

## 4. Conclusions

In summary, this study demonstrates the feasibility and therapeutic potential of a multifunctional ^188^Re-colloid/PTX-micelle hydrogel as a localized treatment strategy for unresectable hepatocellular carcinoma. By integrating a β-emitting radionuclide and a chemotherapeutic agent within a biodegradable and thermosensitive hydrogel matrix, the system enables prolonged intratumoral retention and controlled release of both therapeutic components. Evaluation in a murine hepatic tumor model showed enhanced tumor-localized delivery and improved antitumor efficacy, suggesting a synergistic benefit of combined chemo-radiotherapy. From a translational perspective, the thermosensitive nature of the hydrogel allows minimally invasive intratumoral administration with in situ gelation, which may help limit systemic exposure, while the use of the clinically relevant radionuclide ^188^Re supports the feasibility of future clinical translation. Although further investigations, including large-animal studies, long-term safety assessments, and dose optimization, are required, these findings provide a compelling proof-of-concept for the development of hydrogel-based, localized chemo-radiotherapeutic platforms for the treatment of unresectable solid tumors.

## Figures and Tables

**Table 1:** Quantitative biodistribution of ^188^Re-colloid/PTX-micelle hydrogel after intratumoral administration.

Organ	1 h (%ID)	8 h (%ID)	48 h (%ID)
Blood	0.141 ± 0.077	0.130 ± 0.028	0.011 ± 0.006
Brain	0.100 ± 0.085	0.051 ± 0.014	0.010 ± 0.006
Stomach	0.529 ± 0.202	0.456 ± 0.151	0.016 ± 0.007
Lung	0.557 ± 0.366	0.741 ± 0.747	0.100 ± 0.076
Heart	0.056 ± 0.020	0.036 ± 0.004	0.009 ± 0.002
Liver	0.609 ± 0.382	0.836 ± 0.243	0.377 ± 0.185
Spleen	0.104 ± 0.069	0.128 ± 0.086	0.105 ± 0.042
Kidney	0.228 ± 0.128	0.263 ± 0.041	0.076 ± 0.026
Bone	0.213 ± 0.200	0.173 ± 0.041	0.067 ± 0.063
Muscle	0.049 ± 0.023	0.029 ± 0.010	0.136 ± 0.103
Small intestine	0.056 ± 0.020	0.113 ± 0.045	0.020 ± 0.003
Urinary bladder	0.126 ± 0.080	0.343 ± 0.182	0.043 ± 0.038
Tumor	49.157 ± 11.918	36.628 ± 12.394	36.345 ± 11.461

Data are expressed as percentage of injected dose (%ID) and presented as mean ± SD (*n* = 3 per time point).

## Data Availability

The original contributions presesnted in this study are included in the article. Further inquiries can be directed to the corresponding author.
